# Therapeutic support and functional disability after ischemic stroke: a cohort study

**DOI:** 10.1590/0034-7167-2025-0364

**Published:** 2026-08-21

**Authors:** Brenda Silva Cunha, Mariana de Almeida Moraes, Liane de Assis Campos Medeiros, Bianca Moreira de Santana, Carlos Antônio de Souza Teles Santos, Fernanda Carneiro Mussi

**Affiliations:** I Universidade Federal da Bahia. Salvador, Bahia, Brazil

**Keywords:** Ischemic Stroke, Persons with Disabilities, Cohort Studies, Rehabilitation, Patient Care Team., Accidente Cerebrovascular Isquémico, Personas con Discapacidad, Estudios de Cohortes, Rehabilitación, Grupo de Atención al Paciente.

## Abstract

**Objectives:**

to analyze the association between the therapeutic support received and the functional disability degree between two and three years after ischemic stroke, comparing functional disability level between 90 days and two to three years after the event.

**Methods:**

of the 242 survivors followed, 155 were alive after three years. Sociodemographic and clinical instruments, a telephone protocol, and the Modified Rankin Scale were used. Data were analyzed using chi-square, Fisher’s exact, and McNemar tests (α=0.05).

**Results:**

62.6% presented with asymptomatic/mild dysfunction, and 37.4% with moderate to severe dysfunction. Despite functional improvement, many maintained or worsened their disability (p=0.6219). Follow-up was limited: appointments with neurologists (56.6%), physiotherapists (45.8%), nurses (15%), and secondary prophylaxis (53.6%). Individuals with greater disability had more follow-up, with significant association for physiotherapy.

**Conclusions:**

there were gaps in therapeutic support, despite the persistence or progression of disabilities. Multidisciplinary follow-up is essential to prevent worsening and promote recovery.

## RESUMEN

**Objetivos:**

analizar la asociación entre el apoyo terapéutico recibido y el grado de discapacidad funcional entre dos y tres años después de un ictus isquémico, comparando el nivel de discapacidad funcional entre los 90 días y los dos o tres años posteriores al evento.

**Métodos:**

de los 242 supervivientes a los que se les realizó seguimiento, 155 seguían vivos a los tres años. Se utilizaron instrumentos sociodemográficos y clínicos, un protocolo telefónico y la Modified Rankin Scale. Los datos se analizaron mediante las pruebas de chi-cuadrado, exacta de Fisher y exacta de McNemar (α=0,05).

**Resultados:**

el 62,6% eran asintomáticos o presentaban disfunción leve, y el 37,4% presentaban disfunción moderada a grave. A pesar de la mejoría funcional, muchos mantuvieron o empeoraron su discapacidad (p=0,6219). El seguimiento fue limitado: consultas con neurólogos (56,6%), fisioterapeutas (45,8%), enfermeras (15%) y profilaxis secundaria (53,6%). Las personas con mayor discapacidad tuvieron un mayor seguimiento, con una asociación significativa con la fisioterapia.

**Conclusiones:**

existieron deficiencias en el apoyo terapéutico, a pesar de la persistencia o progresión de las discapacidades. El seguimiento multiprofesional es esencial para prevenir el empeoramiento y promover la recuperación.

## INTRODUCTION

Stroke is among the leading causes of morbidity and mortality in Brazil and worldwide, and the main cause of disability in people over 50 years of age. Among the types of stroke, ischemic stroke is the most frequent^(1,2)^.

Disabilities resulting from an ischemic stroke vary depending on the location and extent of brain damage. They can be temporary or permanent, with degrees of severity ranging from mild to highly disabling^(3)^.

Approximately 75% of affected individuals experience some form of dysfunction, and between 15% and 30% develop severe disability^(4)^. A longitudinal study revealed that more than 30% of individuals required assistance or became completely dependent on someone to perform basic daily tasks, such as eating, dressing, going to the bathroom, or transferring between bed and chair. Concerning the functional disability degree assessment, 41.5% were considered independent, while 29.5% presented moderate dependence, and 29%, severe or total dependence^(5)^.

Due to the impacts of the disease, affected individuals require rehabilitation to minimize sequels and recover, whenever possible, a degree of functional independence. This scenario reveals the importance of ensuring therapeutic support at all levels of the Health Care Network, aiming to minimize the negative repercussions of stroke on quality of life^(6)^.

Considering the disabilities caused by cerebrovascular events, the stroke care pathway provides for the offer of multidisciplinary therapeutic support at various levels of the Health Care Network, adapted to the specific needs of each individual and capable of providing a set of interdisciplinary interventions to support rehabilitation^(7)^.

Therapeutic support aims to maintain functionality-whether physical, sensory, intellectual, psychological, or social-as well as to improve individuals’ interaction with their environment, providing tools that facilitate the achievement of independence and self-determination^(8)^. The need for secondary prophylaxis, rigorous control of risk factors, guidance for health promotion and prevention of new events, and follow-up with regular appointments with a multidisciplinary team is highlighted^(7)^.

The multidisciplinary team practices have the potential to assist in social and work reintegration through guidance, support, and recovery of skills to perform daily tasks, promoting independence^(9)^.

However, even knowing the benefits of therapeutic support after a stroke, access is not always guaranteed, especially in public health systems. Several factors contribute to this limitation, such as the high costs involved in rehabilitation, financial constraints on families, a shortage of qualified professionals in sufficient numbers, and a lack of adequate infrastructure to offer comprehensive therapeutic care^(10)^.

The above reveals the importance of understanding the therapeutic support received by people with ischemic stroke, as well as assessing the disability degree’s evolution over time, which allows for the identification of gaps in care services. In this study, therapeutic support is understood as the set of care actions aimed at continuity of care after an acute event, including secondary prophylaxis, secondary prevention strategies, complication prevention, and rehabilitation. These actions are carried out through appointments with healthcare professionals, such as neurologists, nurses, and physiotherapists, after a stroke.

Knowledge of therapeutic support and disability’s evolution over time, in addition to highlighting individual patient outcomes, can contribute to reflection on the effectiveness of care models tailored to local realities and to the creation of health policies and rehabilitation programs that ensure access to adequate care. Furthermore, understanding the condition of these individuals at different times after a stroke allows for the development of new healthcare technologies.

There is a significant gap in knowledge regarding therapeutic support and functional evolution over longer periods, such as two to three years post-ischemic stroke. This study seeks to fill this gap by providing original data on continuity of care and its relationship with levels of functional disability over time. In the Brazilian context, the findings may highlight inequalities in access to rehabilitation and support more equitable and effective public policies. Internationally, the results contribute evidence for improving follow-up and long-term rehabilitation guidelines, reinforcing the importance of continuous care in stroke survivor populations.

## OBJECTIVES

To analyze the association between the therapeutic support received and the functional disability degree between two and three years after the ischemic stroke, comparing the level of functional disability between 90 days and two to three years after the event.

## METHODS

### Ethical aspects

This study is part of a larger project entitled “*Fatores associados à incapacidade e mortalidade por AVC Isquêmico e aos tempos de acesso ao tratamento*”, approved by the Research Ethics Committee, and follows the guidelines of Resolutions 466/2012 and 580/2018 of the Brazilian National Health Council. The objectives, confidentiality, privacy, right of withdrawal, and clarification regarding the Informed Consent Form were ensured.

### Study design and site

The study was conducted at a public hospital in the state of Bahia, a reference center for the treatment of people with ischemic stroke. It is a prospective cohort study linked to the main project entitled “*Fatores associados à incapacidade e mortalidade por Acidente Vascular Cerebral Isquêmico*”. This study corresponds to phase III of the cohort, which involved following participants from 90 days after the stroke up to three years after the ischemic stroke.

The preparation of this manuscript was guided by the EQUATOR network STrengthening the Reporting of OBservational studies in Epidemiology guidelines^(11)^.

### Sample

Within 90 days of the ischemic stroke, a sample of 242 participants who met the inclusion criteria at baseline was used: a clinically confirmed diagnosis of ischemic stroke recorded in the medical record with a compatible imaging examination and admission to the study site, as well as a minimum age of 18 years. Participants were excluded if they had symptoms that prevented verbal communication and there were no caregivers available to answer the research questions, or if the time since the ischemic stroke was greater than ten days due to the possibility of recall bias. Participants who died between 90 days and three years after the ischemic stroke were also excluded from this study.

**Figure 1:**
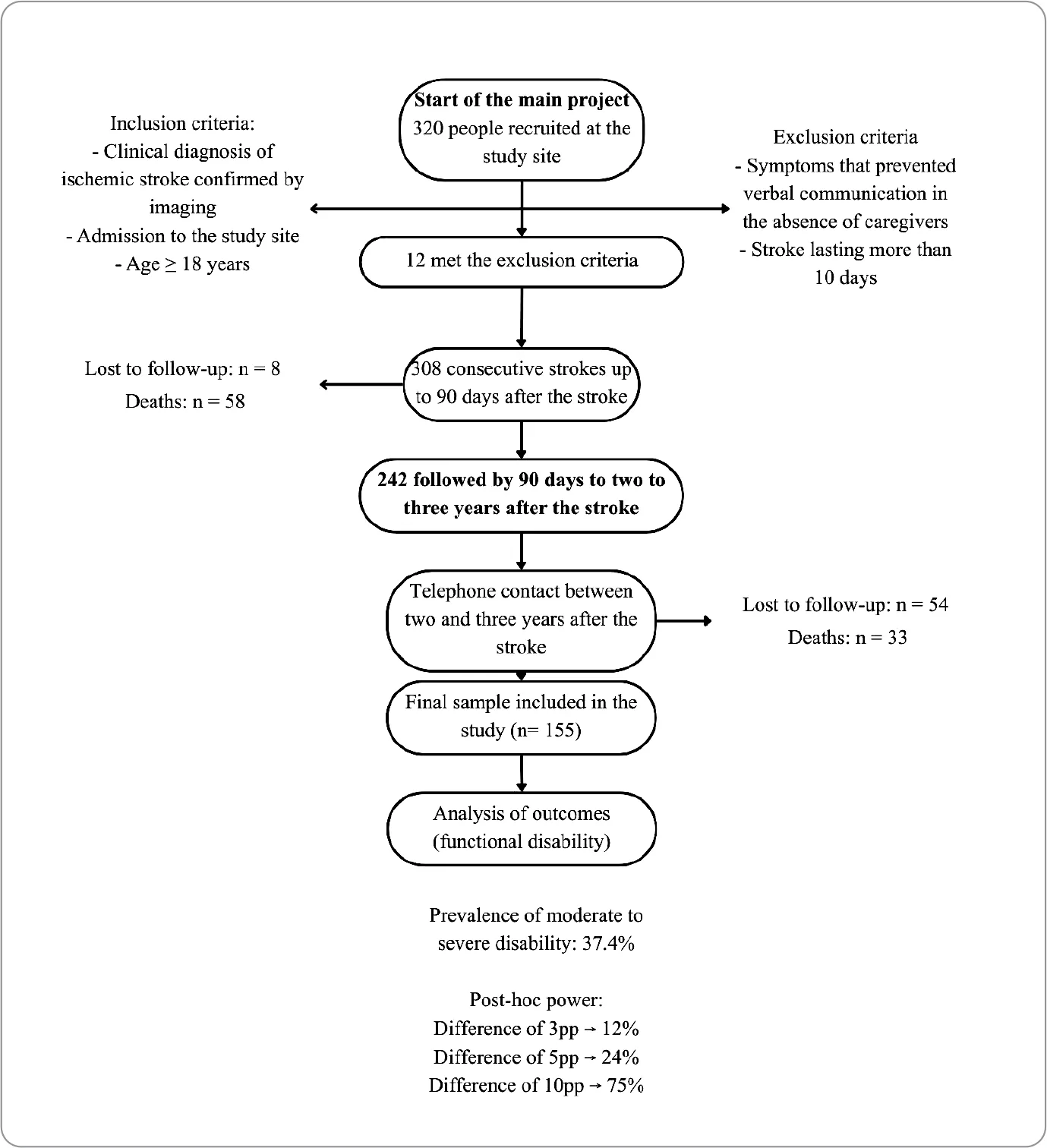
STrengthening the Reporting of OBservational studies in Epidemiology flowchart, Salvador, Bahia, Brazil, 2025

The 242 participants were contacted by telephone between two and three years after the ischemic stroke. Of these, 54 could not be located (loss to follow-up) and 33 died, resulting in a sample of 155 participants for the present study, as shown in Figure 1. Considering the 155 participants included in this study and a prevalence of 37.4% of moderate to severe functional disability, a post-hoc power calculation was performed to detect absolute differences of 3, 5, and 10 percentage points among groups, with a significance level of 5% and a two-tailed test. The estimated statistical powers were 12%, 24%, and 75%, respectively.

### Data collection instruments

In this research, data collected through sociodemographic and clinical characterization instruments of participants in the parent project were used. These instruments included closed-ended, multiple-choice, and semi-structured questions to characterize the sample in relation to sex, race/color, marital status, education level, work activity, monthly family income, age, and place of residence; presence of atrial fibrillation, diabetes, hypertension, dyslipidemia, myocardial infarction, and previous stroke; smoking; admission to the Stroke Unit; thrombolysis; severity of neurological deficit, according to the National Institutes of Health Stroke Scale (NHISS); and time of arrival at the study site after the stroke.

For phase III of this cohort, a telephone call protocol was used, applied during phone calls, to collect data on the occurrence of COVID-19 and new stroke, secondary prophylaxis (self-reported use of anticoagulants and/or antiplatelet drugs), and follow-up appointments with physiotherapists, physicians, nurses, or other healthcare professionals. This protocol also included the Modified Rankin Scale (mRS), the main tool used to assess disability after ischemic stroke. The scale was adapted to Brazilian culture and validated for telephone application^(12)^. This is a Likert-type scale, with scores ranging from 0 to 6, used to measure the functional disability degree: 0 - no symptoms; 1 - no significant disability; 2 - mild disability; 3 - moderate disability; 4 - moderately severe; 5 - severe; and 6 - death. In the present study, the scale was applied only to survivors, disregarding the score of 6 (death)^(13)^. The mRS was administered 90 days and between two and three years after the ischemic stroke (phase III).

### Data collection procedures

Sociodemographic and clinical characterization data were obtained through interviews and review of medical records during the participant recruitment phase from March to October 2019. In situations where a eligible participant lacked the clinical, cognitive, and/or emotional capacity to respond, data were obtained from their caregiver.

Data from the follow-up instrument for participants between two and three years after the ischemic stroke were collected using the telephone call protocol (phase III), via telephone contact, between March 2021 and November 2023. Responses were preferably provided by the participant themselves and, in cases where communication or comprehension was impossible, by a caregiver or family member who accompanied them in daily activities.

Calls were made by two undergraduate students and one master’s student who were properly trained and used smartphones. Three telephone contact attempts were made on different days and times of the week. If a person responsible for contact was unsuccessful, the instrument was passed on to another collaborator so that new attempts could be made, aiming to minimize losses in follow-up. After five unsuccessful telephone attempts, the participant was considered lost to follow-up.

For all participants, the telephone call protocol (phase III) was printed and kept by the interviewers at the time of the call. It contained participants’ registration number corresponding to the one in the database. The records collected on the physical protocol were transferred to a database in the Statistical Package for the Social Sciences (SPSS) version 22.0, and physical protocols were archived for appointment, if necessary.

### Data analysis

For data analysis, the outcome variable functional disability was categorized into two levels: 0 to 2, corresponding to asymptomatic individuals to those with mild disability; and 3 to 5, referring to individuals with moderate to severe disability^(14)^. The independent variables for the functional disability degree were related to the therapeutic support received, all dichotomized as “yes” and “no”: secondary prophylaxis; follow-up by a healthcare professional; appointment with a neurologist; appointment with a physiotherapist; and appointment with a nurse.

Variables related to sociodemographic and clinical characteristics, therapeutic support, and functional disability were analyzed using absolute and relative frequencies.

To analyze the association between the functional disability degree and the therapeutic support received, Pearson’s chi-square test or Fisher’s exact test, the Odds Ratio, and their respective 95% Confidence Intervals (95%CI) were used^(15)^. To compare Rankin scores between 90 days and up to three years after the ischemic stroke, McNemar’s chi-square test was used^(15)^. A statistical significance level of 5% was adopted. Data were processed and analyzed using the SPSS version 22.0.

In relation to missing data, the analyses were conducted considering only the number of valid observations for each variable, with percentages calculated based on the total number of responses obtained (valid cases).

## RESULTS

### Sociodemographic and clinical characteristics

Concerning sociodemographic variables (Table 1), the predominant age range was 60 to 79 years (55.5%), female sex (50.3%), self-declared black race/color (85.7%), married/with partner (52.3%), education up to complete primary school (62.5%), inactive employment status (56.1%), and monthly family income of up to three minimum wages (87.2%).

**Table 1:** Sample sociodemographic characteristics, Salvador, Bahia, Brazil, 2024

Variables	n	(%)
**Sex (n=155)**		
Female	78	50.3
Male	77	49.7
Race/color (n=154)^*^		
White	22	14.3
Black	47	30.5
Brown	85	55.2
**Marital status (n=155)**		
Married/with a partner	81	52.3
Single/widowed/separated/divorced	74	47.7
**Age in years (n=155)**		
Up to 59	53	34.2
60 to 79	86	55.5
≥80	16	10.3
**Education level (n=155)**		
Completed primary education (up to primary school level)	97	62.5
Completed high school (up to high school level)	48	31.0
Up to completed or incomplete higher education	10	6.5
**Employment situation (n=155)**		
With activity	68	43.9
No activity	87	56.1
Income in minimum wage (n=149)^*^		
> 3	19	12.8
> 1 to 3	63	42.2
Up to 1	67	45.0

* *The denominator (n) varies between variables due to missing data.*

As for clinical variables (Table 2), most participants had no history of atrial fibrillation (92.6%), did not have diabetes (76.6%), dyslipidemia (69.1%), previous acute myocardial infarction (91%), previous stroke (73.4%), and were non-smokers (60.6%). Most had not undergone thrombolysis (72.9%), arrived at the study site within 4.5 hours of symptom onset or wake-up stroke (59.4%), and presented with an admission NHISS characteristic of moderate neurological deficit (53.1%). It was identified that 9.5% of participants had COVID-19, and in 11.6%, stroke recurrence occurred between two and three years after the ischemic stroke.

**Table 2:** Sample clinical characteristics, Salvador, Bahia, Brazil, 2024

Variables	n	(%)
Atrial fibrillation (n=148)^*^		
No	137	92.6
Yes	11	7.4
Diabetes (n=154)^*^		
No	118	76.6
Yes	36	23.4
**High blood pressure (n=155)**		
No	37	23.9
Yes	118	76.1
**Dyslipidemia (n=155)**		
No		69.1
Yes	48	30.9
**AMI (n=155)**		
No	141	91.0
Yes	14	9.0
Previous stroke (n=154)^*^		
No	113	73.4
Yes	41	26.6
**Thrombolysis (n=155)**		
No	113	72.9
Yes	42	27.1
**Smoke (n=155)**		
No	94	60.6
Yes	16	10.4
Already smoked	45	29.0
NHISS (n=130)^*^		
Mild deficit	41	31.5
Moderate deficit	69	53.1
Severe deficit	14	10.8
Very severe deficit	6	4.6
COVID-19 (n=154)^*^		
No	141	91.6
Yes	13	8.4
**Recurrence of stroke (n=155)**		
No	143	92.3
Yes	12	7.7

NHISS - National Institutes of Health Stroke Scale; AMI - acute myocardial infarction;

* The denominator (n) varies between variables due to missing data.

### Comparison of the disability degree between 90 days and two to three years after ischemic stroke

Table 3 shows that, of the 43 participants who were asymptomatic 90 days after the stroke, 60.5% maintained this degree between two and three years after the ischemic stroke. However, 39.5% progressed from asymptomatic to worse levels of disability: 20.9% to no significant disability; 11.6% to mild disability; 4.7% to moderate disability; and 2.3% to severe disability.

**Table 3:** Comparison of the functional disability degree at 90 days and between two and three years after ischemic stroke, Salvador, Bahia, Brazil, 2024

Disability degree 90 days after ischemic stroke	Functional disability degree between two and three years after ischemic stroke						
	Asymptomatic n (%)	Without significant disability n (%)	Mild disability n (%)	Moderate disability n (%)	Moderately severe disability n (%)	Severe disability n (%)	Total n (%)
Asymptomatic	26 (60.5)	9 (20.9)	5 (11.6)	2 (4.7)	-	1 (2.3)	43 (100)
Without significant disability	3 (17.6)	7 (41.2)	3 (17.6)	2 (11.8)	-	2 (11.8)	17 (100)
Mild disability	12 (44.5)	6 (22.2)	6 (22.2)	3 (11.1)	-	-	27 (100)
Moderate disability	4 (10.8)	8 (21.6)	4 (10.8)	12 (32.5)	7 (18.9)	2 (5.4)	37 (100)
Moderately severe disability	-	2 (14.3)	-	5 (35.7)	2 (14.3)	5 (35.7)	14 (100)
Severe disability	1 (6.3)	-	-	-	8 (50.0)	7 (43.7)	16 (100)
**Total**	46 (29.9%)	32 (20.8%)	18 (11.7%)	24 (15.6%)	17 (11%)	17 (11%)	154 (100)

Of the 17 participants without significant disability 90 days after the ischemic stroke, 17.6% became asymptomatic; and 41.2% maintained this degree. However, 41.2% experienced a worsening of the disability degree: 17.6% to mild disability; 11.8% to moderate disability; and 11.8% to severe disability.

Of the 27 participants with mild disability 90 days after ischemic stroke, 22.2% maintained this dysfunction; 11.1% progressed to moderate disability; and 66.7% improved their disability degree: 22.2% to no significant disability; and 44.5% to asymptomatic.

Of the 37 participants with moderate disability 90 days after ischemic stroke, 32.5% remained at that level of dysfunction; 24.3% experienced a worsening of the disability degree (18.9% to moderately severe disability; and 5.4% to severe disability); and 43.2% showed improvement in the disability level (10.8% to mild disability; 21.6% to no significant disability; and 10.8% to asymptomatic).

Of the 14 participants who had moderately severe disability, 14.3% maintained this degree; 35.7% progressed to severe disability; and 50% improved their disability degree (35.7% to moderate; and 14.3% to no significant disability).

Finally, of the 16 participants with severe disability 90 days after the ischemic stroke, 43.7% maintained that disability degree; and 56.3% improved (50% progressed to moderately severe disability; and 6.3% became asymptomatic).

McNemar’s chi-square test did not show a statistically significant change (p=0.6219) in the functional disability degree at 90 days and two to three years after the ischemic stroke.

### Therapeutic support received by participants and its association with the disability level

In the mRS assessment of functional disability between two and three years after ischemic stroke, 62.58% were asymptomatic to mildly dysfunctional; and 37.42% had moderate to severe dysfunction.

Most participants were followed up by some healthcare professional (92.3%). Regarding the professional category, most consulted a neurologist (56.6%), did not consult a physiotherapist (54.2%) or a nurse (85%). In addition, 53.6% underwent secondary prophylaxis (53.6%) (Table 4).

**Table 4:** Association between therapeutic support and disability level, Salvador, Bahia, Brazil, 2023

Therapeutic support	Disability level					
	Total n (%)	Asymptomatic to mild dysfunction n (%)	Moderate to severe dysfunction n (%)	*p* value	Odds Ratio	95%CI
**Secondary prophylaxis (n=155)**						
Yes	83 (53.6)	54 (65.1)	29 (34.9)	0.783	0.78	0.36-1.67
No	49 (31.6)	29 (59.2)	20 (40.8)			
Does not know	23(14.8)	14 (60.9)	9 (39.1)			
**Follow-up with a healthcare professional (n=155)**						
Yes	143 (92.3)	87 (60.8)	56 (39.2)	0.122	3.22	0.70-14.9
No	12 (7.7)	10 (83.3)	2 (16.7)			
**Appointment with a neurologist (n= 150)**						
Yes	85 (56.6)	50 (58.8)	35 (41.2)	0.131	1.69	0.83-3.46
No	65 (43.4)	46 (70.8)	19 (29.2)			
**Appointment with a physiotherapist (n=153)**						
Yes	70 (45.8)	34 (48.6)	36 (51.4)	**0.000**	3.34	1.63-6.89
No	83 (54.2)	63 (75.9)	20 (24.1)			
**Appointment with a nurse (n= 153)**						
Yes	23 (15.0)	13 (56.5)	10(43.5)	0.503	1.36	0.58-3.22
No	130 (85.0)	83 (63.8)	47(36.2)			

95%CI - 95% Confidence Interval;

* The denominator (n) varies between variables due to missing data.

Individuals who used secondary prophylaxis had a lower chance of moderate to severe dysfunction compared to those who did not use this preventive measure; however, this association was not statistically significant. Participants who reported follow-up with a healthcare professional, appointment with a neurologist, nurse, and physiotherapist had a higher chance of moderate to severe dysfunction than those who did not have follow-up, with statistical significance found for physiotherapy follow-up. In this case, those who consulted with a physiotherapist were 3.34 (95%CI 1.63-6.89) times more likely to have moderate/severe functional disability than those who did not consult.

## DISCUSSION

This study investigated the association between therapeutic support and the functional disability degree in people affected by ischemic stroke two to three years after the event. The results revealed that most participants presented with clinical symptoms ranging from asymptomatic to mild dysfunction, but more than a third of them still presented with moderate to severe dysfunction. These findings highlight the importance of continuous therapeutic support, both to prevent functional deterioration and to enhance the recovery from existing disabilities.

The finding that participants monitored by healthcare professionals, such as neurologists, nurses, and physiotherapists, had a higher chance of moderate to severe functional disability likely reflects a severity or referral bias. Individuals with greater limitations tend to seek or be referred for ongoing monitoring, which may generate a positive association without implying that the care caused functional worsening. This relationship should therefore be interpreted with caution, especially in healthcare systems with restricted access to rehabilitation, where resources are prioritized for more severe cases. Thus, it is plausible that the observed association is a consequence of the clinical profile of the patients treated, not an adverse effect of multidisciplinary care.

A comparison of the disability degree between 90 days and two to three years after the ischemic stroke revealed that a significant portion of participants with mild, moderate, moderately severe, and severe disability showed functional improvement. However, the proportion of individuals who maintained or worsened their disability degree was also significant. Of particular note is the fact that many of the participants who were initially asymptomatic or had minimal functional impairment progressed to higher levels of disability, and this was precisely the group that had the fewest appointments with physiotherapists, nurses, and neurologists.

Among the various benefits provided to the rehabilitation of people after a stroke, physiotherapy stands out, which aims to improve gait speed through supervised exercises, contributing to an increased quality of life^(16)^. Furthermore, educational interventions by nurses with patients, family members, and caregivers can contribute to improved quality of life, reduced disability, and prevention of new cerebrovascular events^(17)^.

Furthermore, participant clinical characterization stands out, highlighting the presence of risk factors for ischemic stroke, with emphasis on hypertension and recurrence of the event. These findings reinforce the importance of ensuring continuous follow-up through appointments with a nurse, in order to promote appropriate clinical management and prevent future complications. Nurses play a fundamental role in promoting patient autonomy, improving or restoring functionality, encouraging a healthy lifestyle, and mobilizing resources for reintegration into the social, economic, and cultural spheres, always focusing on patient safety^(18-20)^. Regular continuing education sessions, focusing on rehabilitation and prevention of new complications, are essential both during the transition from hospital environments to the home and in continuity of patient follow-up at the Primary Health Care Unit and in home settings^(19)^.

This study revealed not only the low frequency of appointments with nurses, physiotherapists, and neurologists during the rehabilitation period, but also the predominance of these appointments among individuals with moderate to severe disability. Consequently, most asymptomatic participants or those with mild disability did not receive follow-up from these professionals, revealing a lack of access and essential therapeutic support for both rehabilitation and the prevention of new events. It can be hypothesized that the progression of disability levels among initially asymptomatic patients or those with mild dysfunction, over time, is related to the absence of adequate therapeutic support.

The results of this study also highlight the importance of monitoring patient adherence to appointments with the multidisciplinary team. A study conducted in Joinville, Santa Catarina, showed that outpatient follow-up at 30 and 90 days after stroke was associated with better clinical outcomes, with stroke recurrence being the main motivating factor for adherence to appointments^(21)^. Continuous clinical monitoring of stroke survivors, in both the acute and chronic phases, is essential for monitoring new complications and functional evolution, thus contributing to a reduction in hospital readmission rates^(22)^. Furthermore, a randomized clinical trial demonstrated that scheduling medical appointments before hospital discharge, combined with post-discharge telephone follow-up, promoted more frequent appointments^(23)^. Such evidence reinforces the need to establish strategies that ensure effective access to outpatient follow-up after hospital discharge.

The stroke care pathway recommends that outpatient follow-up should begin soon after hospital discharge, with the first appointment ideally taking place between seven and 30 days after the event. The goal is to reassess patients’ clinical and functional conditions, adjust treatment, reinforce therapeutic adherence, identify potential complications early, and make necessary referrals for rehabilitation. Furthermore, it recommends continuous and multidisciplinary follow-up, according to each patient’s needs, aiming at functional rehabilitation and prevention of recurrences. Primary Health Care is responsible for coordinating care over time with the support of a multidisciplinary team^(7)^.

The results of this study revealed significant gaps in the healthcare provided to people with ischemic stroke by the multidisciplinary team, highlighting the need for public policies directed at this population group, as well as effective strategies that ensure rehabilitation in all phases of treatment. In this context, it is important to emphasize that indicators of social inequality were predominant in the sample, such as black race/color, lack of employment, low education level, and low family income. These factors can accentuate vulnerability in the rehabilitation process and have been associated with higher levels of functional disability^(24,25)^.

Low levels of education, for instance, are linked to less knowledge about risk factors, compromising prevention efforts, while unemployment represents a barrier to accessing rehabilitation services due to travel costs and dependence on available social support^(26)^. Furthermore, identifying the majority of participants as being between 60 and 79 years old reinforces a growing demand for home care and other rehabilitation services for older adults, especially those with mobility difficulties. This age group tends to face a lack of family support during recovery and difficulty accessing other support networks, factors that are considered significant challenges in the rehabilitation process and its success^(17)^.

Thus, the need for health policies and public health strategies is evident to ensure that all people with ischemic stroke have equal access to the necessary therapeutic support, regardless of their geographic location or social and economic situation. Furthermore, these policies should enable and invest in research and development to drive innovation in assistive technologies, therapeutic treatments, and rehabilitation techniques, implementing monitoring and assessment systems to measure the effectiveness of rehabilitation programs and ensure the quality of care provided.

### Study limitations

This study has some limitations that should be considered when interpreting the results. Data collection was performed in a single center, which restricts the generalizability of the findings to other populations. Statistical analyses were conducted in a bivariate manner, without adjusting for potential confounding factors, which limits the interpretation of the observed associations.

Furthermore, of the 242 people initially included, there were losses to follow-up and deaths, resulting in a reduced sample size, with the possible introduction of selection bias. Imputation techniques were not applied to deal with missing data, nor was a formal sensitivity analysis performed, which may compromise the robustness of some observed associations. Functional disability assessment was conducted through telephone interviews, and in some cases, with the use of informants. The time interval between the event and data collection may make the data susceptible to recall bias, and the use of informants may also have introduced measurement bias, with possible underestimation or overestimation of outcomes.

Despite these limitations, this is a prospective cohort study with a follow-up of two to three years, addressing a topic that is still little explored in the literature. The study makes a significant contribution to public health by providing evidence that can support the strengthening of existing protocols, as well as guide the formulation of new strategies for longitudinal follow-up of patients in the Health Care Network.

### Contributions to nursing, health, or public policy

This study can support the creation and improvement of individualized care plans for patients after ischemic stroke, considering the different phases of recovery, from hospital discharge to outpatient follow-up, including the early identification of risks and needs for therapeutic support. Furthermore, it can guide the planning of healthcare services, indicating the demand for specific professionals such as physiotherapists, neurologists, nurses, among others, and the need to structure care networks that guarantee continuity of treatment after hospital discharge.

## CONCLUSIONS

Analysis of the therapeutic support received two to three years after ischemic stroke reveals significant gaps in continuity of care, especially regarding multidisciplinary rehabilitation and systematic follow-up. Follow-up appointments, particularly with nurses, were infrequent, and follow-up with physiotherapists was offered only to the most severely disabled patients. Comparison of functional disability levels at 90 days and between two and three years post-ischemic stroke indicated that, although some patients show improvement or stability, a significant proportion remain with significant deficits or worsening disability, reflecting both the limitations of the care provided and the absence of sustained functional and social support strategies.

The study reinforces the need for public policies that prioritize not only acute treatment but also chronic care and rehabilitation, in order to promote the autonomy and quality of life of stroke survivors. Early and continuous rehabilitation for all patients, regardless of the disability degree, remains a challenge in the Brazilian Unified Health System. The study showed the need for advancements in the Health Care Network, ensuring expanded access to specialized treatment units and early and continuous rehabilitation with a multidisciplinary team. Furthermore, effective policies and interventions aimed at health promotion, prevention and control of risk factors for ischemic stroke, and encouraging early seeking of medical care are fundamental to minimize the negative repercussions of the event on the lives of victims and their families.

## Data Availability

The research data are available within the article.
