# Supplementary material for: Therapeutic support and functional disability after ischemic stroke: a cohort study
Source: Rev Bras Enferm. 2026 Aug 21;79:e20250364. doi: 10.1590/0034-7167-2025-0364 (PMC13497370; doi:10.1590/0034-7167-2025-0364)
Supplement: Translation

# Translation: Suporte terapêutico e incapacidade funcional após acidente vascular cerebral isquêmico: estudo de coorte

**DOI:** 10.1590/0034-7167-2025-0364pt

**Authors:** Brenda Silva Cunha, Mariana de Almeida Moraes, Liane de Assis Campos Medeiros, Bianca Moreira de Santana, Carlos Antônio de Souza Teles Santos, Fernanda Carneiro Mussi

**Affiliations:** I Universidade Federal da Bahia. Salvador, Bahia, Brasil

**Keywords:** Acidente Vascular Cerebral Isquêmico, Pessoas com Deficiências, Estudos de Coortes, Reabilitação, Equipe Multiprofissional.

## Abstract

**Objetivos:**

analisar a associação entre o suporte terapêutico recebido e o grau de incapacidade funcional entre dois e três anos após acidente vascular cerebral isquêmico, comparando o nível de incapacidade funcional entre 90 dias e de dois a três anos após o evento.

**Métodos:**

dos 242 sobreviventes acompanhados, 155 estavam vivos após três anos. Utilizaram-se instrumentos sociodemográficos, clínicos, protocolo telefônico e *Modified Rankin Scale*. Dados foram analisados pelos testes qui-quadrado, Fisher e McNemar (α=0,05).

**Resultados:**

62,6% apresentavam-se assintomáticos/disfunção leve, e 37,4%, com disfunção moderada a severa. Apesar da melhora funcional, muitos mantiveram ou agravaram a incapacidade (p=0,6219). O seguimento foi limitado: consultas com neurologistas (56,6%), fisioterapeutas (45,8%), enfermeiros (15%) e profilaxia secundária (53,6%). Indivíduos com maior incapacidade tiveram mais seguimento, com associação significante para fisioterapia.

**Conclusões:**

houve lacunas no suporte terapêutico, apesar da persistência ou progressão de incapacidades. Seguimento multiprofissional é essencial para prevenir agravamentos e promover a recuperação.

## INTRODUÇÃO

O acidente vascular cerebral (AVC) está entre as principais causas de morbimortalidade no Brasil e no mundo, sendo a principal causa de incapacidade em pessoas com mais de 50 anos. Entre os tipos de AVC, o isquêmico é o mais frequente^(1,2)^.

As incapacidades decorrentes de um acidente vascular cerebral isquêmico (AVCi) variam conforme a localização e a extensão da lesão cerebral. Podem ser temporárias ou permanentes, com graus de gravidade que vão de leves a altamente incapacitantes^(3)^.

Cerca de 75% das pessoas acometidas apresentam alguma disfunção, e entre 15% e 30% desenvolvem incapacidade grave^(4)^. Estudo longitudinal revelou que mais de 30% dos indivíduos necessitaram de auxílio ou passaram a depender completamente de alguém para realizar tarefas básicas do dia a dia, como alimentação, vestuário, locomoção até o banheiro ou transferência entre a cama e a cadeira. Em relação à avaliação do grau de incapacidade funcional, 41,5% foram considerados independentes, enquanto 29,5% apresentaram dependência moderada, e 29%, dependência severa ou total^(5)^.

Devido aos impactos da doença, as pessoas acometidas necessitam de reabilitação para minimizar sequelas e recuperar, sempre que possível, um grau de independência funcional. Esse panorama revela a importância de terem assegurado o suporte terapêutico em todos os níveis da Rede de Atenção à Saúde, visando minimizar as repercussões negativas do AVC na qualidade de vida^(6)^.

Considerando as incapacidades provocadas pelo evento cerebrovascular, a linha de cuidado ao AVC prevê a oferta de suporte terapêutico multiprofissional, nos diversos níveis da Rede de Atenção à Saúde, adaptado às necessidades específicas de cada indivíduo e capaz de proporcionar um conjunto de intervenções interdisciplinares para apoiar a reabilitação^(7)^.

O suporte terapêutico tem como objetivo manter a funcionalidade, seja física, sensorial, intelectual, psicológica e social, bem como de melhorar a interação do indivíduo com seu ambiente, fornecendo instrumentos que facilitem o alcance da independência e autodeterminação^(8)^. Salienta-se a necessidade de profilaxia secundária, de controle rigoroso de fatores de risco, de orientações para promoção da saúde e prevenção de novos eventos, e de seguimento em consultas regulares com equipe multiprofissional^(7)^.

As práticas da equipe multiprofissional têm potencial para auxiliar a reintegração social e laboral, por meio da orientação, apoio e recuperação das habilidades para realizar as tarefas cotidianas, promovendo independência^(9)^.

Contudo, mesmo sabendo sobre os benefícios do suporte terapêutico após o AVCi, nem sempre o acesso é garantido, especialmente em sistemas públicos de saúde. Diversos fatores contribuem para essa limitação, como os altos custos envolvidos na reabilitação, restrições financeiras das famílias, escassez de profissionais qualificados em número suficiente e ausência de infraestrutura adequada para ofertar um cuidado terapêutico completo^(10)^.

O exposto revela a importância de se conhecer o suporte terapêutico recebido por pessoas com AVCi, assim como de avaliar a evolução do grau de incapacidade ao longo do tempo, o que permite identificar lacunas nos serviços de atendimento. O suporte terapêutico, no presente estudo, é entendido como o conjunto de ações assistenciais voltadas à continuidade do cuidado após o evento agudo, incluindo profilaxia secundária, estratégias de prevenção secundária, prevenção de complicações e reabilitação. Essas ações são realizadas por meio de atendimentos com profissionais de saúde, como neurologistas, enfermeiros e fisioterapeutas, após um AVC.

O conhecimento do suporte terapêutico e da evolução da incapacidade ao longo do tempo, além de evidenciar os resultados individuais dos pacientes, pode contribuir para a reflexão sobre a efetividade dos modelos de atendimento voltados às realidades locais e para a criação de políticas de saúde e programas de reabilitação que assegurem o acesso a cuidados adequados. Além disso, compreender a condição dessas pessoas, em diferentes momentos após o AVCi, permite o desenvolvimento de novas tecnologias assistenciais em saúde.

Há uma lacuna importante sobre o suporte terapêutico e a evolução funcional em períodos mais longos, como entre dois e três anos pós-AVCi. Este estudo busca preencher essa lacuna, oferecendo dados originais sobre a continuidade do cuidado e sua relação com os níveis de incapacidade funcional ao longo do tempo. No contexto brasileiro, os achados podem evidenciar desigualdades no acesso à reabilitação e subsidiar políticas públicas mais equitativas e eficazes. Em nível internacional, os resultados contribuem com evidências para o aprimoramento das diretrizes de seguimento e reabilitação prolongada, reforçando a importância do cuidado contínuo em populações sobreviventes de AVC.

## OBJETIVOS

Analisar a associação entre o suporte terapêutico recebido e o grau de incapacidade funcional entre dois e três anos do AVCi, comparando o nível de incapacidade funcional entre 90 dias e dois a três anos após o evento.

## MÉTODOS

### Aspectos éticos

O estudo integra o projeto matriz intitulado “Fatores associados à incapacidade e mortalidade por AVC Isquêmico e aos tempos de acesso ao tratamento”, aprovado pelo Comitê de Ética em Pesquisa, e segue as diretrizes das Resoluções n^o^ 466/2012 e n^o^ 580/2018 do Conselho Nacional de Saúde. Garantiu-se a explicação dos objetivos, sigilo, privacidade, direito de desistência e esclarecimento sobre o Termo de Consentimento Livre e Esclarecido.

### Tipo e local do estudo

O estudo foi desenvolvido em um hospital público do estado da Bahia, referência no tratamento de pessoas com AVCi. Trata-se de coorte prospectiva vinculada ao projeto matriz intitulado “Fatores associados à incapacidade e mortalidade por Acidente Vascular Cerebral Isquêmico”. O presente estudo corresponde à fase III da coorte, que correspondeu ao seguimento dos participantes de 90 dias após até três anos do AVCi.

A elaboração deste manuscrito foi norteada pelas diretrizes do *STrengthening the Reporting of OBservational studies in Epidemiology* da rede EQUATOR^(11)^.

### Amostra

Em 90 dias do AVCi, partiu-se de uma amostra de 242 participantes que, no *baseline,* atenderam aos critérios de inclusão: diagnóstico de AVCi confirmado clinicamente e registrado no prontuário, com exame de imagem compatível, admissão no local de estudo e idade mínima de 18 anos. Os critérios de exclusão consistiram em sintomas que impediam a comunicação verbal na ausência de acompanhantes para responder aos questionamentos da pesquisa e tempo de AVCi superior a dez dias devido à possibilidade de viés recordatório. Para este estudo, foram também excluídos os participantes que vieram a óbito entre 90 dias e até três anos do AVCi.

Os 242 participantes foram contatados por ligação telefônica entre dois e três anos do AVCi. Desses, 54 não foram localizados (perdas de seguimento) e 33 vieram a óbito, resultando em uma amostra para o presente estudo de 155 participantes, conforme Figura 1. Considerando os 155 participantes incluídos neste estudo e uma prevalência de 37,4% de incapacidade funcional moderada a grave, foi realizado um cálculo de poder *post-hoc* para detectar diferenças absolutas de 3, 5 e 10 pontos percentuais entre grupos, com nível de significância de 5% e teste bilateral. Os poderes estatísticos estimados foram de 12%, 24% e 75%, respectivamente.

**Figura 1:** Diagrama de fluxo *STrengthening the Reporting of OBservational studies in Epidemiology*, Salvador, Bahia, Brasil, 2025

### Instrumentos de coleta de dados

Nesta pesquisa, foram utilizados dados coletados pelos instrumentos de caracterização sociodemográfica e clínica dos participantes do projeto matriz, que incluíram questões fechadas, de múltipla escolha e semiestruturadas, para caracterizar a amostra em relação ao sexo, raça/cor, estado civil, escolaridade, atividade laboral, renda familiar mensal, idade e local de residência; presença de fibrilação atrial, diabetes, hipertensão, dislipidemia, infarto do miocárdio e AVC prévios; tabagismo; admissão na Unidade de AVC; realização de trombólise; gravidade do déficit neurológico, segundo a *National Institutes of Health Stroke Scale* (NHISS); e tempo de chegada ao local de estudo após o AVC.

Para a fase III desta coorte, foi utilizado um protocolo de ligação telefônica, aplicado durante os telefonemas, para levantar dados sobre a ocorrência da COVID-19 e de novo AVC, profilaxia secundária (autorrelato de uso de anticoagulante e/ou antiplaquetário) e seguimento em consultas com fisioterapeuta, médico, enfermeiro ou outros profissionais de saúde. Esse protocolo incluiu também a *Modified Rankin Scale* (mRS), principal ferramenta utilizada para avaliar a incapacidade após o AVCi. A escala foi adaptada à cultura brasileira e validada para aplicação por telefone^(12)^. Trata-se de escala do tipo Likert, com pontuação de 0 a 6, utilizada para medir o grau de incapacidade funcional: 0 - sem sintomas; 1 - sem incapacidade significativa; 2 - incapacidade leve; 3 - incapacidade moderada; 4 - moderadamente severa; 5 - grave; e 6 - óbito. No presente estudo, a escala foi aplicada apenas aos sobreviventes, desconsiderando o escore 6 (óbito)^(13)^. A mRS foi aplicada em 90 dias e entre dois e três anos após o AVCi (fase III).

### Procedimentos de coleta de dados

Os dados de caracterização sociodemográfica e clínica foram obtidos por entrevista e consulta ao prontuário, na fase de recrutamento dos participantes, no período de março a outubro de 2019. Nas situações em que o participante elegível não tinha condições clínicas, cognitivas e/ou emocionais para responder, os dados foram obtidos com o seu acompanhante.

Os dados do instrumento de seguimento dos participantes entre dois e três anos após o AVCi foram coletados por meio do protocolo de ligação telefônica (fase III), mediante contato telefônico, no período de março de 2021 a novembro de 2023. As respostas foram, preferencialmente, fornecidas pelo próprio participante e, na impossibilidade de comunicação ou compreensão, por um cuidador ou familiar que o acompanhasse nas atividades cotidianas.

As ligações foram realizadas por duas graduandas e uma mestranda que foram devidamente treinadas e utilizaram *smartphones*. Três tentativas de contato telefônico foram realizadas em dias e horários diferentes da semana. Caso a responsável pelo contato não obtivesse êxito, o instrumento era repassado a outra colaboradora, para que novas tentativas fossem realizadas, visando minimizar as perdas no acompanhamento. Após cinco tentativas telefônicas sem sucesso, o participante foi considerado como perda de seguimento.

Para todos os participantes, o protocolo de ligação telefônica (fase III) foi impresso e mantido com as entrevistadoras no momento da ligação. Nele constava o número do registro do participante correspondente ao do banco de dados. Os registros levantados em protocolo físico foram transferidos para um banco de dados no *Statistical Package for the Social Sciences* (SPSS), versão 22.0, e os protocolos físicos foram arquivados para consultas, caso necessário.

### Análise de dados

Para a análise de dados, a variável desfecho incapacidade funcional foi categorizada em dois níveis: 0 a 2, correspondente a indivíduos assintomáticos a com incapacidade leve; e 3 a 5, referente a indivíduos com incapacidade moderada a severa^(14)^. As variáveis independentes para o grau de incapacidade funcional se relacionaram ao suporte terapêutico recebido, sendo todas dicotomizadas em “sim” e “não”: realização de profilaxia secundária; acompanhamento por profissional de saúde; consulta com neurologista; consulta com fisioterapeuta; e consulta com enfermeiro.

As variáveis relacionadas à caracterização sociodemográfica, clínica, ao suporte terapêutico e à incapacidade funcional foram analisadas em frequências absolutas e relativas.

Para analisar a associação do grau de incapacidade funcional com o suporte terapêutico recebido, utilizaram-se o teste qui-quadrado de Pearson ou exato de Fisher, o *Odds Ratio* e respectivos Intervalo de Confiança de 95% (IC95%)^(15)^. Para comparar o Rankin entre 90 dias e até três anos do AVCi, foi empregado o teste qui-quadrado de McNemar^(15)^. Adotou-se significância estatística de 5%. Os dados foram processados e analisados no SPSS, versão 22.0.

Em relação aos dados faltantes (*missing data*), as análises foram conduzidas considerando apenas o número de observações válidas para cada variável, sendo os percentuais calculados com base no total de respostas obtidas (casos válidos).

## RESULTADOS

### Características sociodemográficas e clínicas

Quanto às variáveis sociodemográficas (Tabela 1), predominaram faixa etária de 60 até 79 anos (55,5%), sexo feminino (50,3%), raça/cor autodeclarada negra (85,7%), casados/com companheiro (52,3%), escolaridade até o 1° grau completo (62,5%), situação laboral inativa (56,1%) e renda familiar mensal de até três salários mínimos (87,2%).

**Tabela 1:** Características sociodemográficas da amostra, Salvador, Bahia, Brasil, 2024

Variáveis	n	(%)
**Sexo (n=155)**		
Feminino	78	50,3
Masculino	77	49,7
Raça/cor (n=154)^*^		
Branca	22	14,3
Preta	47	30,5
Parda	85	55,2
**Estado civil (n=155)**		
Casado/com companheiro	81	52,3
Solteiro/viúvo/separado/divorciado	74	47,7
**Idade em anos (n=155)**		
Até 59	53	34,2
60 até 79	86	55,5
≥80	16	10,3
**Escolaridade (n=155)**		
Até 1º grau completo	97	62,5
Até 2º grau completo Até superior completo ou incompleto	48 10	31,0 6,5
**Situação laboral (n=155)**		
Com atividade	68	43,9
Sem atividade	87	56,1
Renda em salário mínimo (n=149)^*^		
> 3	19	12,8
> 1 a 3	63	42,2
Até 1	67	45,0

* *O denominador (n) varia entre as variáveis devido a dados faltantes.*

Em relação às variáveis clínicas (Tabela 2), a maioria dos participantes não tinha história clínica de fibrilação atrial (92,6%), não tinha diabetes (76,6%), dislipidemia (69,1%), infarto agudo do miocárdio prévio (91%), AVC prévio (73,4%) e não era tabagista (60,6%). A maioria não foi submetida à trombólise (72,9%), chegou ao lócus de estudo em até 4,5 horas do início dos sintomas ou *wake up stroke* (59,4%) e apresentou NHISS admissional característico de déficit neurológico moderado (53,1%). Identificou-se que 9,5% dos participantes tiveram COVID-19, e em 11,6%, a recorrência do AVC ocorreu entre dois e três anos do AVCi.

**Tabela 2:** Características clínicas da amostra, Salvador, Bahia, Brasil, 2024

Variáveis	n	(%)
Fibrilação atrial (n=148)^*^		
Não	137	92,6
Sim	11	7,4
Diabetes (n=154)^*^		
Não	118	76,6
Sim	36	23,4
**Hipertensão arterial (n=155)**		
Não	37	23,9
Sim	118	76,1
**Dislipidemia (n=155)**		
Não	107	69,1
Sim	48	30,9
**IAM (n=155)**		
Não	141	91,0
Sim	14	9,0
AVC prévio (n=154)^*^		
Não	113	73,4
Sim	41	26,6
**Trombólise (n=155)**		
Não	113	72,9
Sim	42	27,1
**Fuma (n=155)**		
Não	94	60,6
Sim	16	10,4
Já fumou	45	29,0
NHISS (n=130)^*^		
Déficit ligeiro	41	31,5
Déficit moderado	69	53,1
Déficit grave	14	10,8
Déficit muito grave	6	4,6
COVID-19 (n=154)^*^		
Não	141	91,6
Sim	13	8,4
**Recorrência do AVC (n=155)**		
Não	143	92,3
Sim	12	7,7

NHISS - National Institutes of Health Stroke Scale; IAM - infarto agudo do miocárdio; AVC - acidente vascular cerebral;

* O denominador (n) varia entre as variáveis devido a dados faltantes.

### Comparação do grau de incapacidade entre 90 dias e dois a três anos do acidente vascular cerebral isquêmico

Na Tabela 3, observa-se que, dos 43 participantes assintomáticos aos 90 dias do AVCi, 60,5% assim permaneceram entre dois e três anos do AVCi. No entanto, 39,5% evoluíram de assintomáticos para piores níveis de incapacidade: 20,9% para sem incapacidade significativa; 11,6% para incapacidade leve; 4,7% para incapacidade moderada; e 2,3% para incapacidade grave.

**Tabela 3:** Comparação do grau de incapacidade funcional em 90 dias e entre dois e três anos do acidente vascular cerebral isquêmico, Salvador, Bahia, Brasil, 2024

Grau de incapacidade aos 90 dias do AVCi	Grau de incapacidade funcional entre dois e três anos do AVCi						
	Assintomático n (%)	Sem incapacidade significativa n (%)	Incapacidade leve n (%)	Incapacidade moderada n (%)	Incapacidade moderadamente severa n (%)	Incapacidade grave n (%)	Total n (%)
Assintomático	26 (60,5)	9 (20,9)	5 (11,6)	2 (4,7)	-	1 (2,3)	43 (100)
Sem incapacidade significativa	3 (17,6)	7 (41,2)	3 (17,6)	2 (11,8)	-	2 (11,8)	17 (100)
Incapacidade leve	12 (44,5)	6 (22,2)	6 (22,2)	3 (11,1)	-	-	27 (100)
Incapacidade moderada	4 (10,8)	8 (21,6)	4 (10,8)	12 (32,5)	7 (18,9)	2 (5,4)	37 (100)
Incapacidade moderadamente severa	-	2 (14,3)	-	5 (35,7)	2 (14,3)	5 (35,7)	14 (100)
Incapacidade grave	1 (6,3)	-	-	-	8 (50,0)	7 (43,7)	16 (100)
**Total**	46 (29,9%)	32 (20,8%)	18 (11,7%)	24 (15,6%)	17 (11%)	17 (11%)	154 (100)

*AVCi - acidente vascular cerebral isquêmico.*

Dos 17 participantes sem incapacidade significativa aos 90 dias do AVCi, 17,6% se tornaram assintomáticos; e 41,2% permaneceram nesse grau. Todavia, 41,2% evoluíram com piora do grau de incapacidade: 17,6% para incapacidade leve; 11,8% para incapacidade moderada; e 11,8 para incapacidade grave.

Dos 27 participantes com incapacidade leve aos 90 dias do AVCi, 22,2% mantiveram essa disfunção; 11,1% evoluíram para incapacidade moderada; e 66,7%, melhoraram o grau de incapacidade: 22,2% para sem incapacidade significativa; e 44,5% para assintomático.

Dos 37 participantes com incapacidade moderada aos 90 dias do AVCi, 32,5% permaneceram nesse grau de disfunção; 24,3% tiveram piora do grau de incapacidade (18,9% para incapacidade moderadamente severa; e 5,4% para incapacidade grave); e 43,2% evoluíram com melhora do nível de incapacidade (10,8% para incapacidade leve; 21,6% para sem incapacidade significativa; e 10,8% para assintomáticos).

Dos 14 participantes que tinham incapacidade moderadamente severa, 14,3% permaneceram; 35,7% evoluíram para incapacidade grave; e 50% melhoraram o grau de incapacidade (35,7% para moderada; e 14,3 para sem incapacidade significativa).

Por fim, dos 16 participantes com incapacidade grave aos 90 dias do AVCi, 43,7% mantiveram esse grau de incapacidade; e 56,3% melhoraram (50% evoluíram para incapacidade moderadamente severa; e 6,3% evoluíram para assintomáticos).

O teste qui-quadrado de McNemar não evidenciou mudança estatisticamente significante (p=0,6219) no grau de incapacidade funcional em 90 dias e dois a três anos do AVCi.

### Suporte terapêutico recebido pelos participantes e associação com o nível de incapacidade

Na avaliação da incapacidade funcional entre dois e três anos do AVCi pela mRS, 62,58% estavam de assintomáticos a disfunção leve; e 37,42% tinham disfunção moderada a severa.

A maioria realizou acompanhamento dos participantes com algum profissional de saúde (92,3%). No que tange à categoria profissional, a maioria realizou consulta com um neurologista (56,6%), não a realizou com fisioterapeuta (54,2%) e nem com enfermeiro (85%). Além disso, 53,6% fizeram a profilaxia secundária (53,6%) (Tabela 4).

**Tabela 4:** Associação entre suporte terapêutico e nível de incapacidade, Salvador, Bahia, Brasil, 2023

Suporte terapêutico	Nível de incapacidade					
	Total n (%)	Assintomático a disfunção leve n (%)	Disfunção moderada a severa n (%)	Valor de *p*	*Odds Ratio*	IC95%
**Profilaxia secundária (n=155)**						
Sim	83 (53,6)	54 (65,1)	29 (34,9)	0,783	0,78	0,36-1,67
Não	49 (31,6)	29 (59,2)	20 (40,8)			
Não sabe	23(14,8)	14 (60,9)	9 (39,1)			
**Acompanhamento com profissional de saúde (n=155)**						
Sim	143 (92,3)	87 (60,8)	56 (39,2)	0,122	3,22	0,70-14,9
Não	12 (7,7)	10 (83,3)	2 (16,7)			
**Consulta com neurologista (n= 150)**						
Sim	85 (56,6)	50 (58,8)	35 (41,2)	0,131	1,69	0,83-3,46
Não	65 (43,4)	46 (70,8)	19 (29,2)			
**Consulta com fisioterapeuta (n=153)**						
Sim	70 (45,8)	34 (48,6)	36 (51,4)	**0,000**	3,34	1,63-6,89
Não	83 (54,2)	63 (75,9)	20 (24,1)			
**Consulta com enfermeiro (n= 153)**						
Sim	23 (15,0)	13 (56,5)	10(43,5)	0,503	1,36	0,58-3,22
Não	130 (85,0)	83 (63,8)	47(36,2)			

IC95% - Intervalo de Confiança de 95%;

* O denominador (n) varia entre as variáveis devido a dados faltantes.

Indivíduos que faziam uso de profilaxia secundária apresentaram menos chance de disfunção moderada a severa em comparação aos que não realizavam essa medida preventiva; no entanto, essa associação não foi estatisticamente significante. Os participantes que relataram acompanhamento com algum profissional de saúde, consulta com neurologista, enfermeiro e fisioterapeuta apresentaram mais chance de disfunção moderada a severa do que aqueles que não tiveram acompanhamento, constatando-se significância estatística para o acompanhamento com fisioterapia. Nesse caso, aqueles que consultaram com fisioterapeuta tiveram 3,34 (IC95% 1,63-6,89) vezes mais chance de apresentar incapacidade funcional moderada/grave do que os que não consultaram.

## DISCUSSÃO

O presente estudo investigou a associação entre o suporte terapêutico e o grau de incapacidade funcional de pessoas acometidas por AVCi após um período de dois a três anos do evento. Os resultados revelaram que a maioria dos participantes apresentava quadro clínico variando de assintomático a disfunção leve, mas mais de um terço deles ainda apresentava disfunção moderada a severa. Esses achados ressaltam a importância do suporte terapêutico contínuo, tanto para evitar a deterioração funcional quanto para potencializar a recuperação das incapacidades já instaladas.

A constatação de que participantes acompanhados por profissionais de saúde, como neurologistas, enfermeiros e fisioterapeutas, apresentaram maior chance de incapacidade funcional moderada a grave provavelmente reflete um viés de gravidade ou de indicação. Indivíduos com maiores limitações tendem a buscar ou a ser encaminhados para acompanhamento contínuo, o que pode gerar uma associação positiva sem implicar que o cuidado tenha causado piora funcional. Essa relação deve, portanto, ser interpretada com cautela, especialmente em contextos de sistemas de saúde com acesso restrito à reabilitação, nos quais os recursos são priorizados para casos mais graves. Assim, é plausível que a associação observada seja consequência do perfil clínico dos pacientes atendidos, não de um efeito adverso do cuidado multiprofissional.

A comparação do grau de incapacidade entre 90 dias e dois a três anos após o AVCi revelou que uma parcela significativa dos participantes com incapacidade leve, moderada, moderadamente severa e grave apresentou melhora funcional. No entanto, a proporção de indivíduos que mantiveram ou agravaram seu grau de incapacidade também foi expressiva. Destaca-se, sobretudo, o fato de que muitos dos participantes inicialmente assintomáticos ou com disfunção funcional mínima evoluíram para níveis mais elevados de incapacidade, sendo justamente esse grupo o que menos realizou consultas com fisioterapeutas, enfermeiros e neurologistas.

Entre os diversos benefícios proporcionados à reabilitação de pessoas após um AVCi, destaca-se o acompanhamento fisioterapêutico, que tem como objetivo melhorar a velocidade da marcha por meio de exercícios supervisionados, contribuindo para o aumento da qualidade de vida^(16)^. Além disso, a intervenção educativa do enfermeiro junto a pacientes, familiares e cuidadores pode contribuir para a melhoria da qualidade de vida, redução das incapacidades e prevenção de novos eventos cerebrovasculares^(17)^.

Destaca-se, ainda, a caracterização clínica dos participantes, que evidenciou a presença de fatores de risco para o AVCi, com ênfase na hipertensão arterial e na recorrência do evento. Esses achados reforçam a importância de garantir o acompanhamento contínuo por meio de consultas com o enfermeiro, a fim de favorecer o manejo clínico adequado e prevenir complicações futuras. O enfermeiro desempenha um papel fundamental na promoção da autonomia do paciente, na melhoria ou restauração da funcionalidade, no incentivo a um estilo de vida saudável e na mobilização de recursos para reintegração às esferas social, econômica e cultural, sempre com foco na segurança do paciente^(18-20)^. Encontros regulares de educação permanente, com foco na reabilitação e prevenção de novos agravos, são essenciais tanto na fase de transição do ambiente hospitalar para o domicílio quanto na continuidade do acompanhamento do paciente na Unidade Básica de Saúde e no contexto domiciliar^(19)^.

O presente estudo evidenciou não apenas a baixa frequência de consultas realizadas pelos participantes com enfermeiros, fisioterapeutas e neurologistas durante o período de reabilitação, mas também a predominância desses atendimentos entre indivíduos com grau de incapacidade moderada a grave. Consequentemente, a maioria dos participantes assintomáticos ou com incapacidade leve não recebeu acompanhamento por esses profissionais, revelando uma carência de acesso e de suporte terapêutico essencial tanto para a reabilitação quanto para a prevenção de novos eventos. Pode-se levantar a hipótese de que a progressão do nível de incapacidade entre pacientes inicialmente assintomáticos ou com disfunção leve, ao longo do tempo, esteja relacionada à ausência de suporte terapêutico adequado.

Os resultados deste estudo também ressaltam a importância de monitorar a aderência dos pacientes às consultas com a equipe multiprofissional. Estudo realizado em Joinville, Santa Catarina, evidenciou que o acompanhamento ambulatorial nos períodos de 30 e 90 dias após o AVC esteve associado a melhores desfechos clínicos, sendo a recorrência do evento o principal fator motivador para adesão às consultas^(21)^. A vigilância clínica contínua dos sobreviventes de AVC, tanto na fase aguda quanto na crônica, é essencial para o monitoramento de novos agravos e da evolução funcional, contribuindo, assim, para a redução das taxas de reinternação hospitalar^(22)^. Além disso, ensaio clínico randomizado demonstrou que o agendamento prévio de consultas médicas antes da alta hospitalar, aliado ao acompanhamento telefônico no pós-alta, favoreceu a maior frequência às consultas^(23)^. Tais evidências reforçam a necessidade de estabelecer estratégias que assegurem o acesso efetivo ao seguimento ambulatorial após a alta hospitalar.

A linha de cuidado do AVC preconiza que o acompanhamento ambulatorial seja iniciado logo após a alta hospitalar, com a primeira consulta idealmente realizada entre sete e 30 dias após o evento, com o objetivo de reavaliação das condições clínicas e funcionais do paciente, ajuste do tratamento, reforço à adesão terapêutica, identificação precoce de possíveis complicações e encaminhamentos necessários à reabilitação. Além disso, recomenda que o seguimento seja contínuo e multiprofissional, conforme a necessidade de cada paciente, visando à reabilitação funcional e à prevenção de recorrências, sendo a Atenção Primária à Saúde a responsável por coordenar o cuidado ao longo do tempo com apoio da equipe multidisciplinar^(7)^.

Os resultados do presente estudo evidenciaram lacunas significativas na atenção à saúde de pessoas com AVCi por parte da equipe multiprofissional, destacando a necessidade de políticas públicas direcionadas a esse grupo populacional, bem como de estratégias efetivas que assegurem a reabilitação em todas as fases do tratamento. Nesse contexto, é importante salientar que indicadores de desigualdade social foram predominantes na amostra, como raça/cor negra, ausência de atividade laboral, baixa escolaridade e baixa renda familiar. Esses fatores podem acentuar a vulnerabilidade no processo de reabilitação e têm sido associados a maiores níveis de incapacidade funcional^(24,25)^.

A baixa escolaridade, por exemplo, está relacionada a menor conhecimento sobre os fatores de risco, comprometendo as ações de prevenção, enquanto a ausência de trabalho representa uma barreira de acesso aos serviços de reabilitação, devido aos custos com deslocamento e à dependência do suporte social disponível^(26)^. Além disso, a identificação da maioria dos participantes na faixa etária entre 60 e 79 anos reforça uma demanda crescente de cuidados domiciliares e outros serviços de reabilitação para a população idosa, sobretudo para aqueles com dificuldade de mobilidade. Esse grupo etário tende a enfrentar a falta de suporte familiar na recuperação e a dificuldade de acesso a outras redes de apoio, fatores que são considerados desafios significativos no processo e sucesso da reabilitação^(17)^.

Desse modo, vislumbra-se a necessidade de políticas de saúde e estratégias de saúde pública para garantir que todas as pessoas com AVCi tenham acesso igualitário ao suporte terapêutico necessário, independentemente de sua localização geográfica ou situação social e econômica. Além disso, essas políticas devem viabilizar e investir em pesquisa e desenvolvimento para impulsionar a inovação em tecnologias assistivas, tratamentos terapêuticos e técnicas de reabilitação, implementando sistemas de monitoramento e avaliação para medir a eficácia dos programas de reabilitação e garantir a qualidade dos cuidados oferecidos.

### Limitações do estudo

Este estudo apresenta algumas limitações que devem ser consideradas na interpretação dos resultados. A coleta de dados foi realizada em um único centro, o que restringe a generalização dos achados para outras populações. As análises estatísticas foram conduzidas de forma bivariada, sem ajuste para potenciais fatores de confusão, o que limita a interpretação das associações observadas.

Além disso, das 242 pessoas inicialmente incluídas, ocorreram perdas de seguimento e óbitos, resultando em um tamanho amostral reduzido, com possível introdução de viés de seleção. Não foram aplicadas técnicas de imputação para lidar com dados ausentes, nem realizada análise formal de sensibilidade, o que pode comprometer a robustez de algumas associações observadas. A avaliação da incapacidade funcional foi conduzida por entrevistas telefônicas, e em alguns casos, com uso de informantes. O intervalo de tempo entre o evento e a coleta de dados pode tornar os dados suscetíveis ao viés de memória, e o uso de informantes também pode ter introduzido viés de mensuração, com possível sub ou superestimação dos desfechos.

Apesar dessas limitações, trata-se de coorte prospectiva com seguimento de dois a três anos, abordando uma temática ainda pouco explorada na literatura. O estudo contribui, de forma relevante, para a saúde pública ao fornecer evidências que podem subsidiar o fortalecimento de protocolos existentes, bem como orientar a formulação de novas estratégias de acompanhamento longitudinal de pacientes na Rede de Atenção à Saúde.

### Contribuições para a área da enfermagem, saúde ou políticas públicas

O estudo pode corroborar a criação e o aprimoramento de planos de cuidados individualizados para pacientes após o AVCi considerando as diferentes fases da recuperação, desde a alta hospitalar até o acompanhamento ambulatorial, incluindo a identificação precoce de riscos e necessidades de suporte terapêutico. Além disso, pode nortear o planejamento de serviços de saúde, indicando a demanda por determinados profissionais como fisioterapeutas, neurologistas, enfermeiros, entre outros, e a necessidade de estruturar redes de cuidado que garantam a continuidade do tratamento após a alta hospitalar.

## CONCLUSÕES

A análise do suporte terapêutico recebido entre dois e três anos após o AVCi revela importantes lacunas na continuidade da assistência, especialmente no que se refere à reabilitação multiprofissional e ao acompanhamento sistemático. O seguimento em consultas, especialmente com enfermeiros, foi pouco frequente, e o seguimento com fisioterapeutas foi oferecido aos mais incapacitados. A comparação entre os níveis de incapacidade funcional aos 90 dias e entre dois e três anos do AVCi indicou que, embora alguns pacientes apresentem melhora ou estabilidade, uma parcela significativa permanece com déficits importantes ou deterioração da incapacidade, refletindo tanto os limites do cuidado oferecido quanto a ausência de estratégias sustentadas de suporte funcional e social.

O estudo reforça a necessidade de políticas públicas que priorizem não apenas o tratamento agudo, mas também o cuidado crônico e dirigido à reabilitação, de modo a favorecer a autonomia e qualidade de vida dos sobreviventes de AVC. A reabilitação precoce e continuada para todos os pacientes, independentemente do grau de incapacidade, ainda é um desafio no Sistema Único de Saúde. O estudo mostrou a necessidade de avanços na Rede de Atenção à Saúde, assegurando a ampliação do acesso a unidades de tratamento especializadas e da reabilitação precoce e continuada com equipe multiprofissional. Além disso, são fundamentais políticas e intervenções efetivas voltadas à promoção da saúde, à prevenção e ao controle dos fatores de risco para o AVCi e ao estímulo à procura precoce de atendimento médico para minimizar as repercussões negativas do evento na vida das vítimas e de suas famílias.

## Data Availability

Os dados de pesquisa estão disponíveis no corpo do artigo.
